# Ovarian and uterine alterations following forced swimming: An immunohistochemical study

**Published:** 2016-10

**Authors:** Seyedeh Nazanin Seyed Saadat, Fahimeh Mohammadghasemi, Hannan Ebrahimi, Hanieh Rafati Sajedi, Gelayol Chatrnour

**Affiliations:** 1 *Student Research Center, Guilan University of Medical Sciences, Rasht, Iran*; 2 *Anatomy Department, Faculty of Medicine, Guilan University of Medical Sciences, Rasht, Iran*

**Keywords:** *Swimming*, *Uterus*, *Ovary*, *Estradiol*, *Apoptosis*, *Estrogen receptor*

## Abstract

**Background:**

Physical exercise is known to be a stressor stimulus that leads to reproductive disruption.

**Objective:**

The aim of this study was to evaluate the effect of forced swimming on the uterus and ovaries in mice.

**Materials and Methods:**

Adult mice (N=24) were divided into the following three groups: A, control; B, swimming in water (10^o^C); and C, swimming in water (23^o^C). Swimmers swam for 5 min daily for 5 consecutive days/ wk during 2 wks. An enzyme linked immunosorbent assay was used to determine serum estradiol, follicle stimulating hormone (FSH) and testosterone levels. Immunohistochemistry was performed to study apoptotic cells or estrogen receptor (ER) expression in uterine epithelial cells and ovaries. ANOVA was used for statistical analysis.

**Results:**

Swimming in both groups reduced the serum FSH and estradiol levels (p<0.01) without having a significant effect on the serum testosterone level or percentage of apoptosis in ovarian and uterine tissues (p<0.01) compared with controls. A significant reduction in the number of ERs in the uterus and ovaries, and secondary and graafian follicles were observed in groups B and C compared with controls (p<0.01); however the number of primordial and primary follicles were not significantly changed in the ovaries.

**Conclusion:**

Forced swimming of 2 wks duration reduces the serum levels of FSH and estradiol without having effects on apoptosis in the ovaries or uteri of mice. Over a long period of time, forced swimming may have an adverse effect on fertility.

## Introduction

Physical activity and exercise are body movements that enhance health and prevent diseases, but can increase stress (1). Therefore, exercise is also known as stressor stimulus in humans and animals. The amount of stress is dependent on the type of exercise, intensity and sport duration (1). Stress can cause physiologic alterations in metabolism of, the cardiovascular, respiratory, digestive, renal, and reproductive systems (1). 

In recent years, due to an increased focus on exercises, physician prescriptions, new legislation for women in sports, social changes, and attention to women, women physical activity has increased. Between 6-79% of female athletes have reproductive abnormalities, including delayed menarche, amenorrhea, and oligomenorrhea (2). In addition, women who engage in more physical activity have lower ovulatory activity (3). The exact mechanism underlying reproductive disruption after exercise is not well-defined (4). It has been shown that swimming may adversely affect on fertility (5, 6). Swimming for 15 days significantly reduced spermatid production in male rats (7). However, female swimmers are predisposed to menstrual disorders and delayed puberty (6). 

Any change in sex hormones, such as estradiol, progesterone, follicle stimulating hormone (FSH), and luteinizing hormone (LH), can affect the female reproductive system. Due to expression of androgen receptors (ARs) throughout most stages of follicular development, it seems that androgens, such as the other sex hormones, play a role in folliculogenesis (8, 9).

Due to lack of studies involving the effect of swimming on ovaries and uterus, the study has been designed to evaluate the impact of forced swimming on the uteri and ovaries in mice using immunohistochemistry, histology, and serologic assays.

## Materials and methods

**Animals**

In this experimental study, 24 adult female Balb/c mice (8-10 wks) were housed under standard laboratory condition (12 hr light/dark cycle) with free access to tap water and chow. All procedures have been performed in accordance with Guilan University of Medical Sciences Animal Welfare Legislation. Animals were maintained and handled according to the protocols approved by the Guilan University of Medical Sciences Animal Care and Use Committee. They were purchased from the Razi Institute. 

Mice were divided into three groups: A) control (no swimming); B) swimming in 10^o^C water; and C) swimming in 23^o^C water. Swimmers swam for 5 min daily for 5 consecutive days/wk during 2 wks. Controls were not put inside the water for swimming. They were in their cages with free access to water or food. Forced swimming of all groups was performed during the light phase (10, 11). For having a same condition all animals in their estrus phase were chosen using a vaginal smear (12). 

Animals in groups B and C, were submitted to force swimming in pool (length 100 cm, width 40 cm, depth 60 cm) for 5 min from 12-13 pm daily for 5 days/wk. during 2 wks. Animals were anaesthetized with ketamine 0.4 ml and 0.2 ml xylazine intraperitoneally on day 15. Blood samples were collected for hormone assessment. Their uterus and ovaries were fixed in 10% neutral buffered formalin for 4 days and processed routinely for histological studies. Tissues were sectioned and then stained with Hematoxylin and Eosin. Immunohistochemistry was used for detection of apoptosis and estrogen receptors (alpha ERalpha in uterus and ERbeta in ovary). 

**Hormone measurement**

After sacrificing the mice their venous blood were collected, centrifuged and then the prepared serum was stored in -80^o^C. Serum estradiol, FSH and testosterone levels were measured using an enzyme linked immunosorbent assay (ELISA) kit according to the manufacturer’s instructions (Monobind, USA), (Lsbio, USA) and (Abcam, USA) respectively.

**Histopathologic study**

**Uterus**

5 microscopic field from 5 slides were observed using a light microscope (Olympus, Japan) with a magnification of 400× for each animal. For morphometric assays, 10 uterine luminal epithelial cells were randomly selected. The longitudinal height of the cells were measured using a graded eye piece which connected to the eye lense. Observations were made by an olympus light microscope(Japan) with 1000x magnification field of view( 13). 

**Assessment of folliculogenesis in ovary**

For study of folliculogenesis we used light microscope. Follicles were assessed on the bases of their histologic morphology and were divided into: primordial, primary, secondary and graafian follicles (14).

**Detection of ER**

Dewaxation of the uteine and ovarian tissue’s sections were performed in xylene. Slides then were incubated in EDTA 1 mM in a 98^ₒ^ inside a steamer for 30 min. Then slides were cooled in room and incubated with 3% hydrogen peroxide (Dako). Mouse monoclonal anti-estrogen receptor α and β antibody (Dako 1D5) was applied using a 1/50 dilution for 30 min at room temperature in uterus and ovary sections respectively. Using an Envision Dual link system (Dako, Canada), observations were performed. Counterstaining was done with Hematoxylin. Cells with brown nucleus were showing the presences of ER. 8-10 fields with area of 1×1 mm^2^ from 3 slides were studied, in each mice. In each field number of positive ER cells were counted and divided by both positive and negative cells numbers of both positive and negative cells and were expressed as percentage. 

**Detection of DNA fragmentation or apoptosis**

Immunohistochemistry and TUNEL (terminal deoxynucleotidyl transferase dUTP nick-end labeling) reaction was used for assessing of apoptosis on the basis of the company instructions( Roche, Germany). Cells with dark brown nucleous were considered as apoptotic. 8-10 fields with area of 1×1 mm^2^ from 3 slides were studied, in each animal. In each field number of positive apoptotic cells was counted and then divided by numbers of both positive and negative cells and were expressed as percentage. 

**Statistical analysis**

Data analysis were performed using SPSS version 13.0 for Windows Microsoft 2010. The data were analyzed by the analysis of variance (ANOVA) and Tukey Post Hoc Test. The p<0.05 was considered evidence for statistical significance.

## Results

**Hormone assessments**

**Figure 1:**
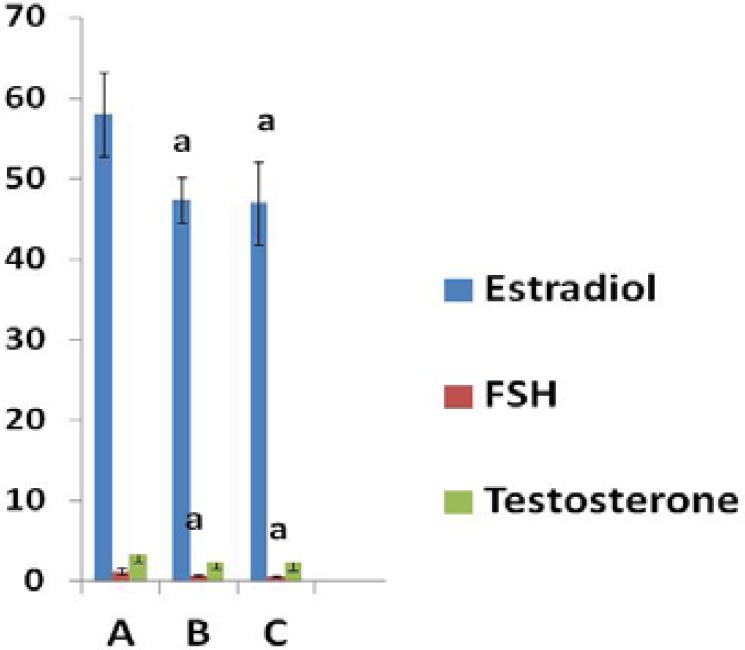
Impact of swimming and temperature on serum levels of FSH , estradiol and testosterone in mouse. Data are expressed as mean± standard deviation. A: control, B: swimming in 10ºC water, C: swimming in 23ºC water. a:significant versus control (p<0.01

The serum levels of estradiol and FSH in controls were 58.01±5.20 pg/ml and 1.24±0.41 mIU/ml, respectively. The estradiol and FSH levels were significantly decreased in all swimming groups compare with controls (p<0.01). The serum testosterone levels in experimental groups were changed but it was not statistically significant. Hormonal level findings in groups are shown in Figure 1.

**Uterus histopathology**

**Figure 2:**
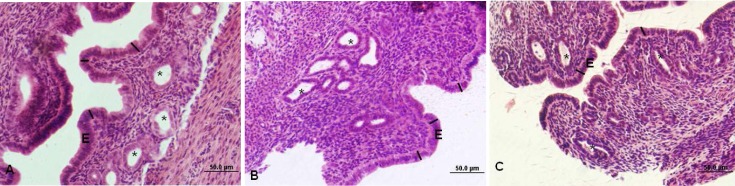
Light photomicrograph of mouse endometrium. A: control, B: swimming in 10ºC water, C: swimming in 23^o^C water, E: Epithelium, stars (*) show endometrial glands. Straight lines show height of epithelium. H&E. Magnification: × 400

The height of epithelial cells in controls was 24.12±1.0 µm. Swimming reduced the height of the epithelial cells in groups B and C compared with the controls (p<0.01) (Table I). Microscopic appearance of endometrium is shown in figure 2.

**Detection of estrogen receptors (ERs) in the uterus**

**Figure 3:**
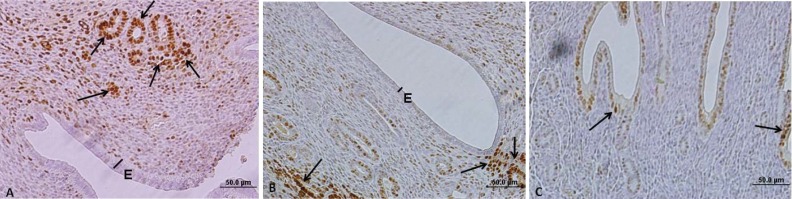
Immunohistochemical staining of ERalpha in mouse endometrium. A: control, B: swimming in 10^o^C water, C: swimming in 23^o^C water. E: Epithelium, Straight lines show height of epithelium. Arrows is showing ERalpha positive cells with brown color. Note the reduced numbers of ER positive cells in swimmers in compare with the controls. Immunohistochemistry staining. Magnification: × 400

The expression of ER-alpha in epithelial cells within control uteri was 22.13±3.43%. Swimming significantly reduced the expression of ER alpha-positive epithelial cells within the uteri in groups B and C (p<0.05); (Table I, Figure 3).

**Detection of apoptosis in the uterus**

The percentage of uterine epithelial cell apoptosis was 1.65±0.63% (Table I). We did not demonstrate a statistical difference between groups with respect to uterine epithelial apoptotic cells compare with the control group. Further, there were no significant differences between groups B and C. 

**Assessment of folliculogenesis in the ovary**

**Figure 4:**
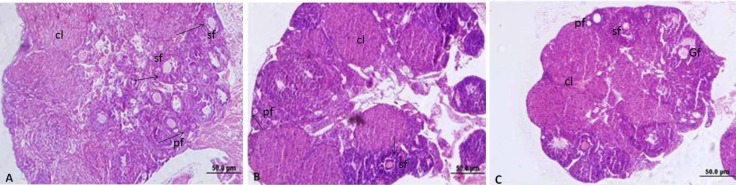
Light photomicrograph of mouse ovary. A: control, B: swimming in 10^o^C water, C: swimming in 23^o^C water. Pf: primary follicle, sf: secondary follicle, Gf: graaf follicle, cl: corpus luteum. Swimming has made a reduced size of ovary especially in group c. H&E. Magnification: × 100

The mean number of primordial and primary follicles per section in controls were14.66±2.16 and 9.18±1.16, respectively. Swimming in groups B and C had no effect on the number of primary or primordial follicles (Figure 4, Table II). The mean number of secondary and graafian follicles in controls were 9.25±0.8 and 2.60±0.96, respectively. Animals in groups B and C had a significantly lower number of both secondary and graafian follicles compared with controls (Figure 4, Table II). 

**Detection of apoptosis in the ovary**

The mean percentage of apoptotic cells in the control ovary was 2.2±0.83. There were no significant differences in the mean percentage of apoptotic cells in groups who were swimming in groups B or C (Table II). 

**Detection of estrogen receptor (ERs) in the ovary**

**Figure 5:**
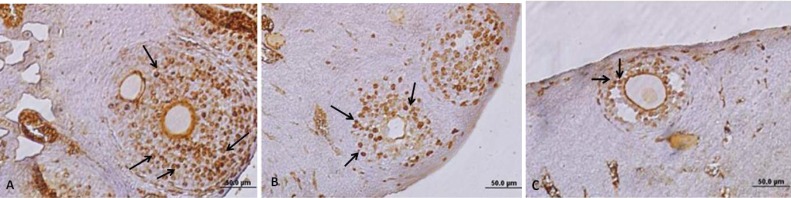
Immunohistochemical staining of ERbeta in mouse ovary. A: control, B: swimming in 10^o^C water, C: swimming in 23^o^C water, Arrows are showing ERbeta positive cells with brown color. Note the reduced numbers of ER positive cells in swimmers in compare with the controls. Immunohistochemistry staining. Magnification: × 400

The mean number of ER-beta in the control ovary was 55.28±8.55. The number of ERs in the groups B and C ovaries were reduced compared with controls (p<0.001). There was a statistical difference in the expression of ERs between group B when compared with group C (p<0.001) (Table II, Figure 5).

**Table I:** Effect of swimming on mouse uterus

**Groups **	**Epithelial height (µm)**	**ER alpha (%)**	**Apoptosis (%)**
A	24.12±1.2	22.13±3.43	1.65±0.63
B	19.12±1.4^a^	15.96±1.58^a^	1.36±0.75
C	19.41±1.6^a^	17.16±2.74^a^	1.28±0.76

Data are expressed as mean± standard deviation**. **A: control B: swimming in water (10^o^C) C: swimming in water (23^o^C) a: significant versus control (p<0.01).

**Table II:** Effect of swimming on mouse ovary

**Groups**	**Primordial. (Fn)**	**Primary. (Fn)**	**Secondary. (Fn)**	**Graaf. (Fn)**	**ERbeta (%)**	**Apoptosis (%)**
A	14.66±2.16	9.18±1.1`6	9.25±0.80	2.60±0.96	55.28±8.55	2.2±0.83
B	15.51±1.98	8.79±0.80	5.06±1.47^a^	1.38±0.76^a^	34.30±5.22^a^	2.21±0.99
C	13.66±1.32	8.01±0.83	4.95±0.33^a^	1.20±0.72^a^	26.40±3.43a	2.35±1.37

Data are expressed as mean± standard deviation. n: number. F: follicle.

A: control B: swimming in water (10^o^C) C: swimming in water (23^o^C) a: significant versus control (p<0.001).

## Discussion

Our study demonstrated that swimming in water for 2 wks at 10 or 23ºC reduces fertility parameters in female mice, including the serum FSH and estradiol levels, as well as ER expression in the uterus and ovary, without an effect on the serum testosterone level and percentage of apoptosis. Therefore, swimming may have an adverse effect on fertility in female mice. The reproductive cycle in mice takes 3-5 days. In humans, the reproductive cycle (i.e., the menstrual cycle) lasts approximately 28 days. Our study was designed for 14 days, which is nearly equivalent to three cycles in humans (15). Uterine changes are completely dependent on ovarian function; therefore any change in the ovary may change uterine function.

Exercise improves ovarian morphology in patients with polycystic ovarian syndrome; however, the effects of swimming on uterine or ovarian morphology have not been examined (16). It is well-known that stress exerts an inhibitory effect on the pituitary-gonadal axis (17). Exercise is a stressor that can affect the serum FSH, progesterone, and estradiol levels (18). In the current study, swimmers had reduced levels of FSH and estradiol in lukewarm (23^o^C) and cold water (10^o^C). It has been shown that cold water results in hydrostatic squeeze, vasoconstriction, and diuresis (19). These changes activate the pituitary for secretion of Adrenocorticotropic hormone (ACTH) and the renin-angiotensin system. Therefore, stress is induced (20). 

Similar to our study, Axelson *et al* showed that forced swimming decreased estrogen and progesterone levels in rats (21). FSH can affect proliferation of follicular cells and regulate estrogen synthesis by granulosa and interna theca cells. The growth of ovarian follicles is fully FSH dependent. In early antral follicles FSH prevents them against cell death (22). Our results showed and confirmed that the numbers of large follicles (secondary and graafian) are reduced in swimmers; these findings can be due to heat stress, which may affect on pituitary gland function and gonadotropins secretion which is necessary for ovary growth and it’s follicles (23).

We conclude that the reduced level of FSH in all swimmers resulted in a reduced number of follicular cells in the ovary, and therefore a reduced secretion of estradiol. Our study also showed that swimming reduced the epithelial height in the uterus. Uterine epithelial growth is regulated by estrogen (24). Therefore, one of the probable mechanisms for reducing the epithelial height in swimmers is a reduced serum estradiol level, as shown in our study. The serum free testosterone level did not differ between the groups. 

Androgens have important roles in folliculogenesis process (9). It especially affects the large follicles maturity (Graaf follicles). Because they express androgen receptors in their cumulus cells in a high amounts (8, 9). Theca cells are responsible for production of androgens in follicles. Granulosa cells, through the action of P450 aromatase, converts androgens into estrogens, estrone, and 17β-estradiol (25). In the present work, swimming significantly decreased ER-alpha and -beta in the uterus and ovary. Estrogen hormone action is mediated by two estrogen receptors (ER-alpha and-beta), which act as ligand-modulated transcription factors (26). 

The two receptors bind 17β-estradiol (E_2_) with high affinity. ER-alpha and -beta have different patterns of expression in the uterus and ovary. In rodents, cycling hormone levels regulate the activity of ER-alpha. Studies have shown that E_2_ increases both ER-alpha and the progesterone receptor (26). Apoptosis is a programmed cell death process that takes places in different cells in both embryonic and adult periods (27). The probable mechanism responsible for exercise-induced apoptosis includes increased glucocorticoid hormone, intracellular calcium concentration, and reactive oxygen species (28). 

We could not find an increase in the apoptosis rate in the ovary or uterus in swimmer mice. In this regard, it has been shown that exercise involving different types of cells causes apoptosis, inhibits apoptosis, or has no effect on apoptosis (29, 30). Heat stress is an environment that drives body temperature above the set-point temperature. Heat stress has adverse effects on reproductive function in males and females. Thus, heat stress changes follicular growth, steroid secretion, oocyte development, and gene expression (23). Heat stress in dairy cows reduces the estradiol level and LH receptor expression (31). 

Little is known about the impact of swimming on the female reproductive system; however, the mechanisms by which heat stress changes reproductive hormones are not well understood. Increased corticosteroid secretion and oxidative levels may be another probable cause (32). Overall, this study showed the adverse effects of forced swimming on female reproductive organs. A limitation of this study was the lack of cellular or molecular mechanisms for study of sex hormone changes and their receptors on the ovary or uterus. Further studies involving adrenal gland function and stress effect on the hypothalamo-pituitary-gonadal axis are warranted. 

## Conclusion

In conclusion, our study showed that swimming in water 5 days per week for duration of 5 min over 14 days in female mice changes the serum FSH and estradiol levels without having an effect on the serum testosterone level or apoptosis percentage in the ovary or uterus. Swimming reduced ER-alpha expression in the uterus and ER-beta expression in the ovary. Over a long period of time, forced swimming may have an adverse effect on fertility. 

## note

***This article extracted from M.D. thesis. (Hannan Ebrahimi)***

## References

[1] Contarteze R, Manchado F, Gobatto C, Mello M (2007). Biomarkers of stress in rats exercised in swimming at intensities equal and superior to the maximal estable lactate phase. Revista Brasileira de Medicina do Esporte.

[2] Warren M, Perlroth N (2001). The effects of intense exercise on the female reproductive system. J Endocrinol.

[3] Olive D (2010). Exercise and fertility:an update. Curr Opin Obstet Gynecol.

[4] Gudmundsdottir SL, Flanders WD, Augestad LB (2009). Physical activity and fertility in women: the North-Trøndelag Health Study. Hum Reprod.

[5] Jana K, Dutta A, Chakraborty P, Manna I, Firdaus SB, Bandyopadhyay D (2014). Alpha-lipoic acid and N-acetylcysteine protects intensive swimming exercise-mediated germ-cell depletion, pro-oxidant generation, and alteration of steroidogenesis in rat testis. Mol Reprod Dev.

[6] Constantin N, Warren M (2013). Menstrual dysfunction in swimmers: a distinct entity. J Clin Endocrinol Metab.

[7] Mingoti GZ, Pereira RN, CM M (2003). Fertility of male adult rats submitted to forced swimming stress. Braz J Med Biol Res.

[8] Walters KA (2015). Role of androgens in normal and pathological ovarian function. Reproduction.

[9] Yoshihiro J Ono, Akiko Tanabe, Yoko Nakamura, Hikaru Yamamoto, Atsushi Hayashi, Tomohito Tanaka (2014). A Low-Testosterone State Associated with Endometrioma Leads to the Apoptosis of Granu-losa Cells. Plos One.

[10] Nakao C, Ookawara T, Kizaki T, Oh-Ishi S, Miyazaki H, Haga S (2000). Effects of swimming training on three superoxide dismutase isoenzymes in mouse tissues. J Appl Physiol.

[11] Nagaraja HS, Jeganathan PS (1999). Forced swimming stress-induced changes in thephysiological and biochemical parameters inalbino rats. Indian J Physiol Pharmacol.

[12] Seyed Saadat S, Mohammadghasemi F, Khajeh Jahromi S, Homafar M, Haghiri M (2014). Melatonin protects uterus and oviduct exposed to nicotine in mice. Interdiscip Toxicol.

[13] Sarani SA, Ghaffari-Novin M, Warren MA, Dockery P ID C (1999). Morphological evidence for the 'implantation window' in human luminal endometrium. Hum Reprod.

[14] Mohammadghasemi F, Khajeh Jahromi S, Hajizadeh H, Homafar MA N S (2012). The Protective Effects of Exogenous Melatonin on Nicotine-induced Changes in Mouse Ovarian Follicles. J Reprod Infertil.

[15] Caligioni C (2009). Assessing reproductive status/stages in mice. Curr Protoc Neurosci.

[16] Qiu S, Wu C, Lin F, Chen L, Huang Z, Jiang Z (2009). Exercise Training Improved Insulin Sensitivity and Ovarian Morphology in Rats with Polycystic Ovary Syndrome. Horm Metab Res.

[17] Chand D, Lovejoy DA (2011). Stress and reproduction: controversies and challenges. Gen Comp Endocrinol.

[18] Jurkowski J, Jones NL, Walker C, Younglai E, Sutton JR (1978). Ovarian hormonal responses to exercise. J Appl Physiol Respir Environ Exerc Physiol.

[19] Tipton M, Bradford C (2014). Moving in extreme environments: open water swimming in cold and warm water. Extrem Physiol Med.

[20] Ganong W (1977). The renin-angiotensin system and the central nervous system. Fed Proc.

[21] Axelson J (1987). Forced swimming alters vaginal estrous cycles, body composition, and steroid levels without disrupting lordosis behavior or fertility in rats. Physiol Behav.

[22] Dorrington J, Bendell JJ, Khan S (1993). Interactions between FSH, estradiol-17β and transforming growth factor-β regulate growth and differentiation in the rat gonad. J Steroid Biochem Mol Biol.

[23] Hansen P (2009). Effects of heat stress on mammalian reproduction. Philos Trans R Soc Lond B Biol Sci.

[24] Cooke PS, Buchanan DL, Young P, Setiawan T, Brody J, Korach K (1997). Stromal estrogen receptors mediate mitogenic effects of estradiol on uterine epithelium. Proc Natl Acad Sci U S A.

[25] Gleicher N, Weghofer A, Barad D (2011). The role of androgens in follicle maturation and ovulation induction: friend or foe of infertility treatment?. Reprod Biol Endocrinol.

[26] Wang H, Eriksson H, Sahlin L (2000). Estrogen receptors alpha and beta in the female reproductive tract of the rat during the estrous cycle. Biol Reprod.

[27] Faraone-Mennella MR, Marini M, Ferone A, Cacace O, Liguoro A, Margonato V (2010). Physical exercise activates the poly(ADP-ribosyl)ation system in rat testes. J Biol Regul Homeost Agents.

[28] Mooren F, Lechtermann A, Völker K (2004). Exercise-induced apoptosis of lymphocytes depends on training status. Med Sci Sports Exerc.

[29] Jin JJ, Ko IG, Kim SE, Shin MS, Kim SH, Jee YS (2014). Swimming exercise ameliorates multiple sclerosis-induced impairment of short-term memory by suppressing apoptosis in the hippocampus of rats. J Exerc Rehabil.

[30] Peters EM, Van Eden M, Tyler N, Ramautar A, Chuturgoon AA (2006). Prolonged exercise does not cause lymphocyte DNA damage or increased apoptosis in well-trained endurance athletes. Eur J Appl Physiol.

[31] Ozawa M, Tabayashi D, Latief T, Shimizu T, Oshima I, Kanai Y (2005). Alterations in follicular dynamics and steroidogenic abilities induced by heat stress during follicular recruitment in goats. Reproduction.

[32] De Rensisa F, Scaramuzzib RJ (2003). Heat stress and seasonal effects on reproduction in the dairy cow-a review. Theriogenology.

